# Personality Traits Influence on Perception and Hesitancy Towards COVID-19 Vaccination Among Tertiary Care Dental Hospital in Delhi: A Cross-Section Study

**DOI:** 10.7759/cureus.34048

**Published:** 2023-01-21

**Authors:** Radhika Gupta, Vikrant Mohanty, Aswini Balappanavar, Puneet Chahar, Kavita Rijhwani, Sonal Bhatia

**Affiliations:** 1 Public Health Dentistry, Maulana Azad Institute of Dental Sciences, New Delhi, IND; 2 Public Health Dentistry, Maulana Azad Institute of Dental Sciences, Delhi, IND

**Keywords:** perception, hesitancy, vaccination, personality traits, covid-19 retro

## Abstract

Background

In the COVID-19 pandemic, vaccination is recognized as a global public-health goal for preventing, containing, and stopping transmission. But the reluctance of people to receive safe and recommended available vaccines (i.e., vaccine hesitancy) was a growing concern. One of the key elements that influence how people are perceived and their reluctance to get the COVID-19 vaccine was found to be personality traits. Hence, the aim of the study was to assess the influence of personality traits on perception and hesitancy towards COVID-19 vaccination among patients attending tertiary dental care hospitals in Delhi.

Methodology

A cross-sectional questionnaire survey was conducted among a sample of 322 participants aged 15-70 years attending the outpatient department of a public sector tertiary care dental hospital in New Delhi. Data was collected over a two-month period using a validated self-administered questionnaire which recorded demographic variables, individual perceptions, hesitancy towards COVID-19 vaccination, and personality traits [using 20-item mini international personality item pool (IPIP)]. Descriptive analysis followed by a Chi-square test and correlation test was applied.

Results

A total of 322 participants were contacted among which 300 participants (93%) responded which comprised 157 males (52.3%) and 143 females (47.7%). Dominant agreeableness personality shows a statistically significant positive correlation with individual perception (r=0.124, p=0.032) while a negative correlation with vaccine hesitancy (r= -0.146, p= 0.011). Among reasons for vaccine hesitancy, fear of side effects showed a significant association with personality traits (p= 0.018).

Conclusion

This study concluded that personality trait (dominant agreeableness) was an important factor in shaping individual perception and hesitancy towards COVID-19 vaccination.

## Introduction

The COVID-19 pandemic an emerging and re-emerging infection surfaced in China in December 2019. Globally it poses a major population health threat, affecting over 335.2 million people, and continues to impose enormous rates of morbidity and mortality [1]. WHO declared COVID-19 a “Public Health Emergency of International Concern” on 30th January 2020 and a “Pandemic on 11th March 2020”[1]. It has totally changed the view of the healthcare system more than ever by highlighting the importance of risk factors, the limits on access to healthcare systems, and the difficulties involved in the development, safety, and accreditation of new drugs and vaccines [2-3]. To curb this pandemic, Government has proposed various preventive measures despite which infection continues to promulgate [4]. For ages, vaccination has been proven to be an effective solution in the management of infectious disease outbreaks such as smallpox, [5] Ebola virus, [6], etc. Further, with the introduction of WHO's Expanded Programme of Immunization in 1974 and the Global Alliance for Vaccination and Immunization in 2000, vaccination was found to have an enormous contribution to global health [7].

During the COVID-19 pandemic, vaccination is recognized as a panoramic public-health goal for preventing, containing, and ceasing transmission. India began the COVID-19 vaccination drive in January 2021. Till now, India has administered vaccines to over 1.62 billion people including first doses (68.53% population) and fully vaccinated (50.76% population) of the currently-approved vaccines [8]. India initially approved the Covishield (Oxford-AstraZeneca vaccine) [9] and Covaxin (by Bharat Biotech) [10] and followed by Sputnik V (Dr. Reddy's Laboratories) [11].

But the reluctance of people to receive safe and recommended available vaccines (i.e., vaccine hesitancy) was a growing concern even before this COVID-19 pandemic. In 2019, vaccine hesitancy was reported as a top ten global health threat by World Health Organization (WHO) [12]. In India, COVID-19 vaccination also faces many challenges in its acceptance. Still, half of the population (47.4%) is not vaccinated. Evidence has shown many explicit reasons for non-vaccination for many diseases including contextual factors such as socioeconomic, cultural, environmental, behavioral, or political factors; personal factors including social/peer/familial influence, or specific beliefs related to religion or social norms; and vaccination-related issues such as concern about its need, side effects, or safety related to the vaccine. [13-14] 

One such factor which is very less explored is personality traits. As personality traits are stable characteristics of an individual which differ in their pattern of thinking, feeling, and behaving in different situations. To describe the diverse and broader understanding of individual personality, the five-factor model is most commonly used. It includes neuroticism (i.e., feelings of anxiety, anger, guilt, frustration), extraversion (i.e., manifested in outgoing, talkative, energetic), conscientiousness (i.e., vigilant, careful, organized, aim for achievement), openness to experience (i.e., intellectual curiosity, perceptive, creative, reflective), and agreeableness (i.e., kind, cooperative, sympathetic, trustworthy) [15-16]. Though, previous research has explored the influence of personality traits on attitude toward health behavior [17-18]. Also, very little evidence has explored the association of different personality traits with acceptance of COVID-19 vaccination [19].

As the world has continued to witness an unprecedented surge in COVID-19, vaccination is likely to be the promising solution to flatten the curve of infection. However, the negative attitude of people toward COVID-19 vaccination is a growing concern in many countries including India. Hence, identifying, and addressing the factors towards non-acceptance of COVID-19 vaccination can be an essential step to ensure the rapid and requisite uptake of an eventual vaccine, and develop risk communication strategies for future pandemics as well. Hence, the present study was the first attempt in India to assess the influence of personality traits on an individual’s perception and hesitancy toward COVID-19 vaccination in a tertiary dental care hospital in Delhi.

## Materials and methods

A cross-sectional questionnaire study was conducted among individuals aged 15-70 years over a period of two months i.e., March 2021-April 2021 attending the outpatient department of a public sector tertiary care dental hospital in New Delhi. 

The present cross-sectional was conducted in accordance with the principles included in the Declaration of Helsinki (7th revision, 2013) and in accordance with local statutory requirements. Written informed consent was obtained from all the participants. Participants were informed about the purpose of the study, the data protection rights, and the right to refuse participation in the study or to terminate the participation without reasoning or penalty. The hospital administration was informed about the research objectives and permission was obtained prior to starting the study. The collected data were summarized and reported in the aggregate and used only for scientific purposes. The survey methodology was applied with minimal risk or harm to study participants.

The sample size (n) was determined based on the formula Z2pq/L2 where the prevalence (P) of the willingness of individuals to receive COVID vaccination was found to be 70% [19]. The calculated sample size was 322 participants. Patients who were willing to participate and give their consent were included in the study. 

There were two main study tools. A self-administered questionnaire, which had questions pertaining to sociodemographic details, COVID-19 status and source of information, individual perception, and reasons for hesitancy towards COVID-19 vaccination.

Secondly, the big five personality traits were measured using a standardized, validated 20-item mini-international personality item pool (IPIP) [20] with four items per trait. Participants responded on a five-point Likert scale ranging from one (strongly disagree) to five (strongly agree). Eleven items were reverse coded and total items were averaged to create indexes of each trait. A higher score signified that an individual had a more prominent personality trait.

As part of this study, the self-developed questionnaire and mini IPIP were translated (English to Hindi) by a language expert and back-translated (Hindi to English) for translation reliability. No significant differences in responses were noted between those surveyed in different languages.

The data was collected by a single trained investigator. This survey underwent a pilot testing procedure before the implementation of the final study on 20 subjects in the outpatient department of a public sector tertiary care dental hospital in Delhi for the purpose of training and calibration of examiners and to ensure the reliability and validity of the questionnaire.

The face and content validity of the questionnaire was carried out by a team of 10 public health experts. The scale content validity index (S-CVI) was calculated to be 0.88. The set of questions was assessed by other authors for comprehension, adequacy, and linguistics, and rephrased till the understanding was clear and the questions were determined to be appropriate.

The data was entered into a digital spreadsheet with proper coding and statistically analyzed using SPSS (IBM Corp. Released 2012. IBM SPSS Statistics for Windows, Version 21.0. Armonk, NY: IBM Corp). A test for normal distribution was conducted using Kolmogorov-Smirnov test and distribution was found to be skewed. Frequencies and descriptive statistics of various variables were identified and tabulated. Following this, Chi-Square Test was applied to the data. The responses to the questionnaire were dichotomized for statistical analysis and better interpretability. Correlation tests were done to assess the relationship between perception and hesitancy towards COVID vaccination with personality traits. Significant statistical outcomes were set at p-value < 0.05 and a 95% confidence interval.

## Results

Among 322 participants, 300 (93%) participants comprising 157 males (52.3%) and 143 females (47.7%) were responded and included in this study. However, a 7% (n=22) non-response rate of the participants was observed.

The majority of the sample i.e., 48.3% (n=45) were 20-34 years old, 41.7% (n=125) had completed graduation, 28.3% (n=85) reported to have income in the range of 29,000-49,000 rupees per month and 37.7% (n=113) were having arithmetic skill jobs. Around 58% (n=174) of study samples belonged to the lower middle-class status. The most common source of information related to COVID-19 was found to be television news (49.3%, n=148) followed by social media (26%, n=78). Most of the study participants had never experienced COVID-19 Infection (86%, n=259) (Table 1).

**Table 1:** Distribution of sociodemographic variables and COVID-19 related status in study subjects

Demographic Factors	n	%
Age groups		
19 years	45	15
20-34 years	145	48
35-44 years	60	20
45-60 years	41	13.7
>60 years	9	3
Gender		
Males	157	52.3
Females	143	47.7
Education		
Illiterate and primary school	2	0.7
Middle school	14	4.7
High school	56	18.7
Intermediate	31	10.3
Graduate	125	41.7
Professionals	72	24.0
Income		
10,000-29,972	48	16
29,972-49,961	85	28.3
49,962-74,755	65	21.7
74,755-99,930	60	20
99931-199861	28	9.3
>199862	14	4.7
Occupation		
Unemployed	22	7.3
Unskilled and semi-skilled	14	4.7
Skilled workers	59	19.7
Arithmetic skill jobs	113	37.7
Semi-professionals	56	18.7
Professionals	36	12
Socio-economic status		
Upper	12	4
Upper middle	109	36.3
Lower middle	174	58
Lower	5	1.7
COVID status (past 6 months)		
Positive	18	6
Negative	282	94
Source of information		
News	148	49.3
Social media	78	26
Healthcare providers	7	2.3
Family & friends	11	3.7
All	115	38.3

Table 2 shows the distribution of the big five personality traits among study participants. The majority of the participants had prominent agreeableness personality (28.3%, n=85), followed by conscientiousness (22%, n=66), imaginative (15.5%, n=47), neuroticism (6.3%, n=19), and least by extraversion personality (5.3%, n=16). Study participants also showed various combinations (22.3%, n=67) of more than one personality trait. Among them, 19% of individuals were having a combination of conscientiousness and agreeableness personality, 11% showed a combination of neuroticism and imaginative, and 2% of all individuals have shown all types of traits.

**Table 2:** Distribution of different big five personality traits among study subjects SD= Standard Deviation

Big Five Personality traits	n (%)	Median (IQR)
Agreeableness	85(28.3)	3(3.25-2.5)
Concioustiousness	66(22)	3.5(4-3.25)
Extraversion	16(5.3)	3.5 (4-3)
Neuroticism	19(6.3)	3 (3.5-2.75)
Imaginative	47(15.6)	3.25 (3.75-3)
Combinations	67(22.3)	

When the perception of study participants regarding COVID-19 vaccination was assessed, it was found that 93% (n=279) participants were aware of COVID-19 vaccination, whereas 74% (n=224) were ready to receive COVID-19 vaccination. Whereas 62% (n=186) participants supported the need to follow precautionary measures as laid by the Government of India even after getting a vaccination to prevent the further spread of infection (Table 3).

**Table 3:** Perception regarding COVID vaccination

Questions	Agree	Neither agree nor disagree	Disagree	p value
	n(%)			
Awareness regarding COVID-19 vaccination	279 (93%)	4 (1.3%)	17 (5.6%)	<0.05*
Willingness to receive the COVID-19 vaccine	224 (74%)	42 (14%)	48(16%)	<0.05*
No need to follow precautionary practices after vaccination	86 (28%)	28 (9.6%)	186 (62%)	<0.05*

**Figure 1:**
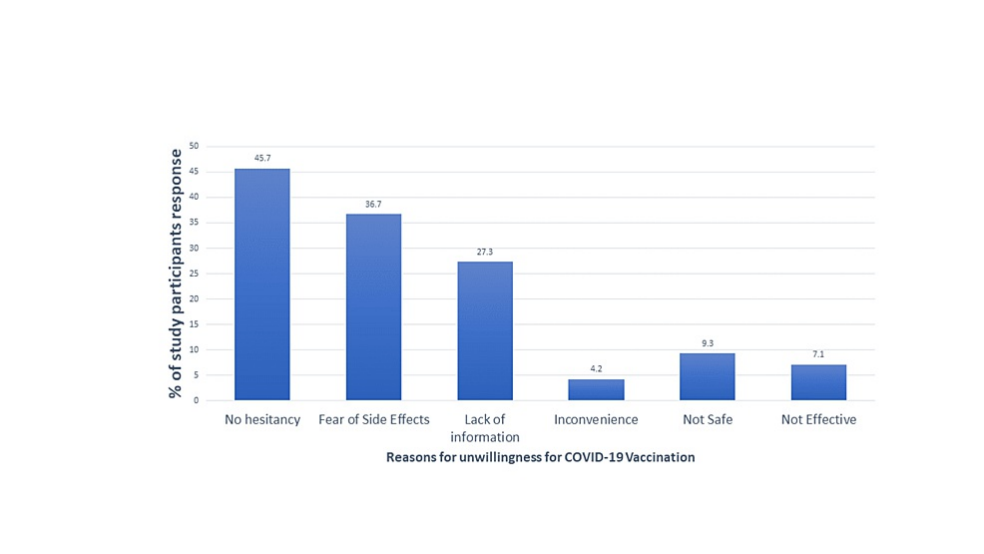
Percentage distribution of reasons for hesitancy toward COVID-19 vaccination

Figure 1 illustrates the reasons for hesitancy toward COVID-19 vaccination. The majority of study participants i.e., 45% reported no vaccine hesitancy while 36% had fear of side effects followed by a lack of information among 27% of participants. Concerned about the effectiveness of the vaccine was seen among 7% of total participants.

Table 4 shows the correlation between personality traits and with perception and hesitancy of the study population toward COVID-19 vaccination. Dominant agreeableness personality shows a statistically significant positive correlation with perception (r= 0.124, p value= 0.03), while a negative correlation was found with vaccine hesitancy (r_s_= -0.146, p value=0.01).

Among the reasons for hesitancy towards COVID-19 vaccination, fear of side effects showed a significant association with all five personality traits including combinations (p=0.01) (Table 5).

**Table 4:** Correlation between personality traits with perception and hesitancy towards COVID-19 vaccination rs= spearman correlation coefficient

Personality Traits	Individual Perception Regarding COVID-19 Vaccination			Hesitancy Towards COVID-19 Vaccination	
	r_s_	p-value		r_s_	p value
Extraversion	-0.63	0.28		0.010	0.86
Agreeableness	0.124	0.032		-0.146	0.011
Conscientiousness	0.093	1.06		-0.36	0.538
Neuroticism	0.27	0.64		-0.027	0.64
Imaginative	-0.026	0.65		0.026	0.65

**Table 5:** Association of personality traits with individual perception and reasons for hesitancy toward COVID-19 vaccination

Variable	Personality Traits (n)						Chi square value	p value
	Extraversion	Agreeableness	Conscientiousness	Neuroticism	Imaginative	Combinations		
Fear of side effects	6	22	28	13	18	27	13.46	0.018
Lack of information	5	23	23	4	13	17	2.310	0.81
Inconvenience	0	2	4	2	2	3	3.766	0.58
Not safe	2	7	12	1	3	4	7.878	0.15
Not effective	2	7	7	0	2	4	4.11	0.53
No hesitancy	9	47	28	6	20	32	5.637	0.34
Awareness regarding COVID-19 vaccination	14	79	61	17	44	64	1.82	0.87
Willingness to receive COVID-19 Vaccine.	10	65	52	13	30	53	5.93	0.31
Need to follow precautionary practices after vaccination.	11	51	39	10	29	47	3.286	0.66

## Discussion

COVID-19 vaccine acceptance and uptake pose an enormous challenge worldwide. Understanding the perception and hesitancy towards vaccination will help in identifying the gaps related to hesitancy and will help healthcare workers and health policymakersto address this issue through different behavioral and risk communication strategies. This present study provides concrete evidence about the influence of personality traits on individual perception towards COVID-19 vaccination among the Indian Population.

The key findings of the study showed that 93% of the study participants were aware of COVID-19 vaccination whereas 74% showed a willingness to receive the vaccination. This is in contrast with the findings of the study conducted in Mysuru, India which reported comparatively lower willingness (60%) towards COVID-19 vaccination. [21]

These findings are in the line with the studies conducted in France, [22] Denmark, [23] Australia, [24]. Whereas contrast, findings from UK, [25], and China, [26] were reported to have 63%, 64%, and 91% willingness to receive COVID-19 Vaccine. These variations in the findings among different countries might be attributed due to the influence of varied socio-economic, cultural, political, and geographical characteristics.

Although, WHO has clearly advised that even after receiving the COVID-19 vaccination, precautions should be continued to follow in order to end this pandemic and stop new variants from emerging [27]. In our study, only 62% of study participants were found to be agreed that there is a need to follow preventive measures even after receiving the vaccination. This might be due to the lack of awareness about remerging nature of COVID-19 infection.

Our study substantiates the fact that individuals with predominant agreeableness personalities have significantly positive perceptions towards COVID-19 vaccination. These findings were consistent with the previous study conducted by Lin et al. in the United States [19]. This might be due to the fact that people having dominant agreeableness personality are more trusted, cooperative, motivated, and understands the need of the situation [15]. Thus, can be considered one of the most important personalities to shape individual behavior toward prevention and vaccination.

Vaccine hesitancy has been reported as a cause of the resurgence of vaccine-preventable disease [12]. Understanding the reasons for vaccine hesitancy has always been important for public health and now vital in this COVID-19 as well as future pandemics. For instance, the measles, mumps, and rubella (MMR) vaccine in 2019 in the United States was considered unsafe by 10% of individuals. Thus, similar findings have been reported for COVID-19 vaccination. Our study reported 54% of study participants showed hesitancy towards vaccination due to various reasons such as Fear of side effects (36.7%), Lack of information (27.3%), Inconvenience (4.2%), Not safe and effective (16.4%). Although lower vaccine hesitancy was reported among other countries such as European Nations (26%) [22], Ireland, and the United Kingdom (31-35%) [18]. The reason for higher hesitancy towards vaccination might be because of the timings of the study as it was conducted immediately after the initiation of the vaccination drive in India so many people were hesitant to receive a vaccination at that timeframe.

The vaccine hesitancy towards COVID-19 vaccination was found to be negatively correlated with a dominant agreeableness personality and this is in line with the findings of the study conducted in the United Kingdom and Ireland [19].

Information-seeking behavior was found to be dependent on individual personality and situational requirements [28]. Therefore, reliable, accurate information and counseling need to be conducted to change the perception of individuals with specific personality traits. Public health messages and Institutional Ethical Committee (IEC) materials can be tailor-made according to the personality traits that are more likely to be vaccine-hesitant or resistant.

The development of personality can be used as a predictive tool in public health practice to not only curb this pandemic but also in future emergencies. The present study can help policymakers to identify the probable reasons for vaccine hesitancy and so plan future public health interventions. It can be included as one of the components in the behavior changes communication process to create more positive understanding among the target population. Also, personality can be added as one of the parameters in health policy planning. Policymakers must quicken dialogue and advance the community networks' development, leveraging and strengthening existing local channels that guide decision-making, such as community leaders, teachers, and popular social media.

However, this study was challenged by a number of inherent limitations that were beyond the control of the investigator. As these were self-reported data from self-selected participants, thus we assessed individual declarations rather than their actual behavior. Also, like other survey-based research, there would be social desirability bias in the study sample when reporting for personality traits, as well as their perception of COVID-19 vaccination. Albeit, the study benefits from its large sample but has limited generalizability due to the specific study settings. The present study has only assessed personality traits and did not include dark traits [29] or situation perceptions [30] of an individual. Lastly, this data was collected immediately after the commencement of the vaccination drive in India, so the rate of willingness and hesitancy towards vaccination will be affected by these social circumstances.

## Conclusions

The study concluded that the personality factor (dominant agreeableness) was directly influencing the individual perception toward vaccine hesitancy. This highlights the importance of shaping the health behavior of the target population. Though a large population sample was aware of vaccination but was reluctant to adopt it due to various reasons such as fear of side effects, lack of information, inconvenience to take, and not being safe and effective. Hence these barriers also needed to be identified and efforts should be made to utilize these factors in adopting a positive health attitude.
